# From Stop to Start: Tandem Gene Arrangement, Copy Number and *Trans*-Splicing Sites in the Dinoflagellate *Amphidinium carterae*

**DOI:** 10.1371/journal.pone.0002929

**Published:** 2008-08-13

**Authors:** Tsvetan R. Bachvaroff, Allen R. Place

**Affiliations:** Center of Marine Biotechnology, University of Maryland Biotechnology Institute, Baltimore, Maryland, United States of America; Centre for DNA Fingerprinting and Diagnostics, India

## Abstract

Dinoflagellate genomes present unique challenges including large size, modified DNA bases, lack of nucleosomes, and condensed chromosomes. EST sequencing has shown that many genes are found as many slightly different variants implying that many copies are present in the genome. As a preliminary survey of the genome our goal was to obtain genomic sequences for 47 genes from the dinoflagellate *Amphidinium carterae*. A PCR approach was used to avoid problems with large insert libraries. One primer set was oriented inward to amplify the genomic complement of the cDNA and a second primer set would amplify outward between tandem repeats of the same gene. Each gene was also tested for a spliced leader using cDNA as template. Almost all (14/15) of the highly expressed genes (*i.e.* those with high representation in the cDNA pool) were shown to be in tandem arrays with short intergenic spacers, and most were *trans*-spliced. Only two moderately expressed genes were found in tandem arrays. A polyadenylation signal was found in genomic copies containing the sequence AAAAG/C at the exact polyadenylation site and was conserved between species. Four genes were found to have a high intron density (>5 introns) while most either lacked introns, or had only one to three. Actin was selected for deeper sequencing of both genomic and cDNA copies. Two clusters of actin copies were found, separated from each other by many non-coding features such as intron size and sequence. One intron-rich gene was selected for genomic walking using inverse PCR, and was not shown to be in a tandem repeat. The first glimpse of dinoflagellate genome indicates two general categories of genes in dinoflagellates, a highly expressed tandem repeat class and an intron rich less expressed class. This combination of features appears to be unique among eukaryotes.

## Introduction

Taken together dinoflagellates are important primary producers in oceans, estuaries, and lakes, and also create environmental problems with disruptive and destructive blooms. Compared to most eukaryotes dinoflagellates have a number of unusual features, including large amounts of DNA [1], unusual DNA bases [2], the absence of nucleosomes [3], [4] and a proclivity for endosymbiosis. While most dinoflagellates are pigmented with the distinctive carotenoid peridinin, some dinoflagellates also harbor plastids derived from haptophytes [5], diatoms [6], green algae [7] and cryptophytes [8]. Since gene transfer is required for plastid retention, dinoflagellates would seem to have an active gene transfer mechanism. An example of gene transfer is that most organellar genes have been transferred to the nucleus with few genes retained in either the mitochondrial [9] or chloroplast genomes [10].

A combination of EST surveys and targeted gene sequencing suggest high copy number and tandem gene arrangement for many dinoflagellate genes. Detailed studies of luciferase [11], luciferin binding protein [12], light harvesting complex [13], rubisco [14], and the peridinin-chlorophyll a-protein [15], [16] indicated high copy number and low intron density. A few genes such as luciferase and peridinin-chlorophyll a-protein, appear to be in tandem arrays with many individual copies of the same gene arrayed together. For example in *Lingulodinium polyedrum*, the peridinin-chlorophyll a-protein gene is present as ∼5000 identical copies per genome with the copies linked by short intergenic spacers [15]. However, when other abundantly expressed genes are compared, a large number of synonymous substitutions were noted between different ESTs in *L. polyedrum*, *Amphidinium carterae* and *Karenia brevis* [10], [17]. These results suggest high copy number for many genes and a certain degree of diversity between these copies.

The control of expression in dinoflagellates is also enigmatic, with studies showing mRNA quantity does not reflect protein levels even in strongly circadian genes such as luciferase [12]. Microarray studies also suggest little change in the RNA pool under different conditions [17], [18]. Recently, *trans*-splicing of dinoflagellate mRNAs was demonstrated where a specific 22 base oligonucleotide, here called the *trans*-spliced leader, was found at the 5′ end of a heterogeneous group of mRNAs [19], [20]. Presumably the oligonucleotide is added by a *trans*-splicing reaction since the gene donating the *trans*-spliced leader has been sequenced. The *trans*-splicing system in dinoflagellates may provide a level of regulation in addition to transcription.

The curious features of the nucleus, active gene transfer, tandem gene arrangement, and *trans*-splicing in dinoflagellates, make it clear that the basic cell biology of dinoflagellate differs from other eukaryotes. We used a simple PCR approach to obtain genomic (120 kb) and cDNA (98 kb) sequences for a suite of 47 different genes from the dinoflagellate *Amphidinium carterae*. The genomic arrangement, *trans*-splicing, polyadenylation, and intron sites of these genes were tested. The strategy was designed explicitly to avoid potential problems with large insert genomic libraries caused by the size, gene arrangement, and unusual DNA bases of the dinoflagellate genome. As a starting point we assumed all the genes were arranged in tandem, so that ‘outwardly’ directed primers would produce PCR products bridging tandem repeat genes. This is similar to an inverse PCR strategy, although in this case the template is unmodified genomic DNA. Similarly we assumed that the mRNAs for these genes would be *trans*-spliced, so that PCR with a gene specific reverse primer and a generic *trans*-spliced leader forward primer using cDNA template would successfully amplify full-length message. *A. carterae* was chosen because the genome size is relatively small for a dinoflagellate at 5.9 pg per cell [1], and because the plastid is pigmented with the typical dinoflagellate pigment peridinin. The organellar and nuclear genomes have been particularly well studied with sequencing of what constitutes both organellar genomes [9], [10], [21], [22], and an already completed EST project [10] gives a good sequence database from which to work. Genes were selected from high, medium, and low expression levels from the earlier EST project with an emphasis on readily identifiable genes. A more detailed study of two genes, actin and a polyketide synthase gene was used for comparative context, since these genes appear to represent the extremes of expression and genomic arrangement. The results allow for a description of different categories of genes in dinoflagellates and shed light on gene arrangement, family size, and regulation in these enigmatic protists.

## Results

Using PCR, the genomic arrangement, *trans*-splicing sites, polyadenylation sites, intron locations, and copy number were tested for 47 genes (Table 1). In each case, tandem arrangement was tested using primers designed to bridge between copies, and conventional inward primers to amplify genomic copies corresponding to selected cDNAs. The results are summarized in Table 1. In addition the genomic arrangement of two genes, actin and *psbO*, from *Karlodinium veneficum* was demonstrated.

**Table 1:** Genes selected for this survey.

Name	Source^a^	# of EST^b^	Tandem repeat^c^	*Trans*-splice site^d^	Genomic^e^	Intron^f^
ESV polysaccaride binding		26	EU710608			
Actin	host	24	EU742749-95	EU742719-48	EU742749-95	1
hsp70	host	24	EU710589-92	EU742860		
hsp90	host	23	EU710606-7	EU742821	EU876701^g^	25
Elongation factor 1 alpha	host	21	EU710609-12	EU742869	EU742870	3
Adenosylhomocysteinase	host	21	EU710593		EU742862	0
psbO	plastid	18	EU876703	EU742841-55	EU876703^g^	9
S-adenosylmethionine synthase	host	17	EU710591-2	EU742861		
Alpha tubulin	host	15	EU710595	EU742865		1
Hypothetical plastid protein	plastid	15	EU710594	EU742864	EU742863	2
GAPDH	host	12	EU710596-7	EU742866	EU742867	0
Beta tubulin	host	12		present		
Basic nuclear protein	host	11	EU710586-8	EU742858	EU742858	1
GAPDH	plastid	10	EU710598-604	EU742868		
Peridinin chlorophyll a protein	plastid	10	EU710605	EU742804	EU742805	0
Glutamate semialdehyde synthase	plastid	8		EU742797	EU742796	0
Translation initiation factor 4A	host	6		present	EU742806	0
Elongation factor 2	host	5	EU876702	present	EU742807	1
Fructose-1,6-bisphosphate aldolase	plastid	5		EU742800		
Nucleoredoxin	host	4		present	EU742803	0
Fructose 1,6-bisphosphate aldolase	host	4		present	EU742802	1
Ribonucleotide reductase	host	4		present	EU742801	0
Ascorbate peroxidase	host	4			EU742799	0
Replication protein	host	4			EU742798	0
atpH	plastid	4	EU710584-5	EU742825-40	EU710584-5	1
U2 snRNP auxiliary factor	host	3				
Alpha-1,2-mannosyltransferase	host	3		present	EU742808	0
ChlD	plastid	3				
Vacuolar-ATPase Beta subunit	host	2		EU742809		
Aspartate carbamoyltransferase	host	2				
UDP-Glucose dehydrogenase	host	2		EU742810	EU742811	0
DNA topoisomerase	host	2		present	EU742812	0
Small nuclear ribonucleoprotein E	host	2		EU742814	EU742813	6
Violaxanthin deepoxidase (1564)	plastid	1				
Violaxanthin deepoxidase (1563)	plastid	1			EU742815	0
Rubisco	other	1				
Translation initiation factor 3 subunit 8	host	1		present	EU742816	8
RNA binding motif	host	1			EU742819	3
StrG	other	1		EU742817	EU742818	1
Ketoyl-Reductase like	host	1				
Cysteine desulfurase	host	1		present	EU742820	0
RNA binding	host	1		present		
Axoneme protein	host	1				
pfsec61	host	1				
PKS- Ketoyl Reductase domain	host	1	no	present	EU742823	18
Mitochondrial	mitochondrion	20			EU742822	0
Totals for 47 genes		359	16	22	31	

a Inferred evolutionary source of gene.

b Number of ESTs in a previous survey [10].

c Tandem repeat genes are repeats of the same gene in a series demonstrated with PCR and sequencing using outwardly directed primers.

d PCR and sequencing with a general spliced leader primer and a gene specific reverse primer. If this PCR was unsuccessful, but was amplified from the same cDNA using gene specific primers then the entry is marked “present”.

e If genomic amplification using inwardly directed primers, followed by sequencing produced a genomic complement to the EST.

f The number of introns found by comparing the expressed and amplified portions of the genomic sequences.

g The intergenic amplicon was used as a proxy for a forward genomic amplicon, since the intergenic amplicon covered almost the entire coding region of these genes.

### Tandem repeats

**Figure 1:**
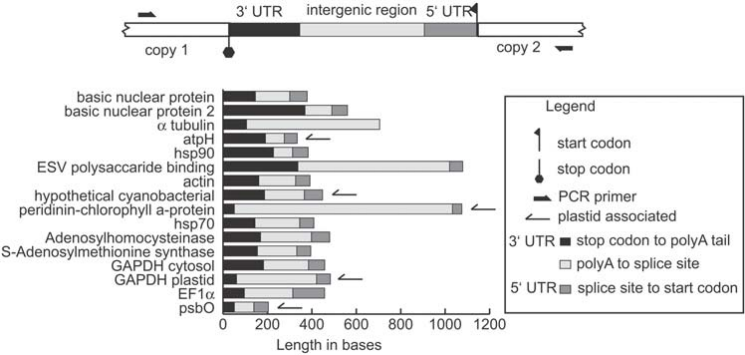
Tandem array intergenic regions from *Amphidinium carterae*. At the top of the figure is a schematic proposed arrangement, applying to all the genes in the survey. The outward primer orientation is designed to amplify between two tandemly arranged copies of the same gene. At the bottom the lengths of the different regions, corresponding to the 3′ UTR, an intergenic region, and the 5′ UTR are plotted for each of the different genes. The *trans*-splicing sites were inferred by comparing cDNA and genomic amplicons. The 3′ UTR was measured by comparing ESTs and genomic sequences. In the case of α tubulin the splice acceptor site is unknown. Although elongation factor 2 was shown to be in a tandem array arrangement, the end of the coding region is ambiguous, and so this gene is not included.

**Figure 2:**
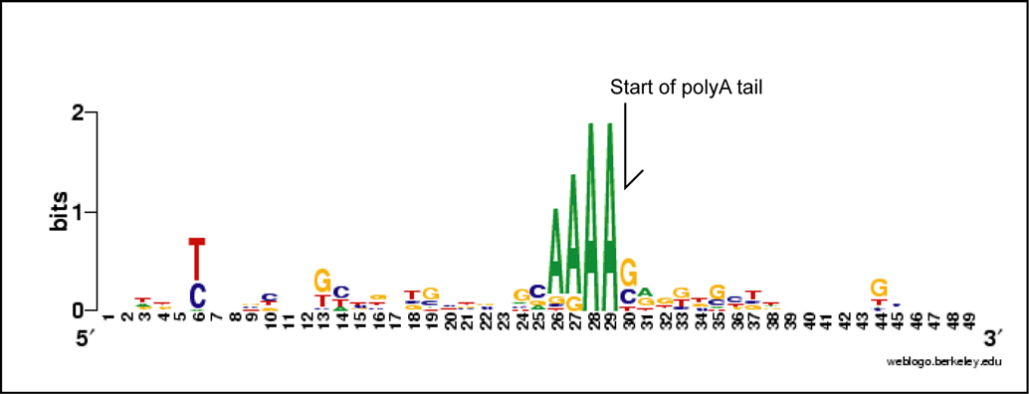
A sequence logo showing the putative polyadenylation sequence for dinoflagellates. This logo is based on an alignment of the intergenic spacer genomic amplicons of 25 different intergenic spacer sequences from 14 genes. The polyadenylation site was inferred by comparing ESTs to the genomic sequence. The same motif is present in *Karlodinium veneficum* and *Lingulodinium polyedrum*.

Sixteen genes were shown to be in tandem repeats based on successful amplification between gene copies, sequencing, and comparisons with the expressed versions of the gene. The intergenic region can be divided into three components (Figure 1): first the stop codon to the polyadenylation site, or genomic complement of the 3′ UTR of the mRNA, second an intergenic region between the polyadenylation site and the *trans*-splicing site (excised from the mature mRNA), and finally the splicing site to the start codon, or the 5′ UTR. Comparing the intergenic regions for different genes, the average size of the 5′ UTR was 165 bases (range 51–369), the 3′ UTR was 72 bases (range 42–143), and the region between the 3′ and 5′ UTRs was an average of 283 bases (range 85–982; Figure 1). Comparing cDNAs to genomic copies revealed a polyadenylation sequence motif that is simply AAAAG/C in the genome at the exact polyadenylation site in *A. carterae* (Figure 2) and *K. veneficum*. Genomic versions of luciferin binding protein (L06908) and the peridinin-chlorophyll a-protein gene (GPU93077) from GenBank for *L. polyedrum* were also compared to expressed sequences and the same polyadenylation motif was present.

In the case of the basic nuclear protein two types of clones were sequenced. The smaller clone contained one intergenic region, as was seen for the other genes, but a second, larger clone contained a complete open reading frame and associated intergenic regions inserted between the two gene copies. The open reading frame sandwiched between copies of the basic nuclear protein encoded another basic nuclear protein from a distinct family. While the different copies of one family were ∼85% identical at the nucleotide level, this gene shared 75% nucleotide identity (134/177) and 61% amino acid identity (48/78) with the flanking genes.

A *trans*-spliced leader like sequence was found in both genomic and cDNA versions of several 5′ UTRs. In the peridinin-chlorophyll a-protein genomic sequence a very close match (21/22 bases) to the *trans*-spliced leader sequence was seen right at the 5′ RNA end. Either the leader is *trans*-spliced exactly at the site of an identical sequence, or the mRNA is initiated from, or truncated to the correct oligonucleotide without the need for *trans*-splicing.

### Intron mapping with inwardly directed primers

Genomic amplicons derived from conventional inwardly directed primers allowed for intron mapping when compared to the cDNA (Table 1). In some sense, these amplicons were designed as a control to compare with the results of outwardly directed primer sets. First, many genes were readily amplified and sequenced with this method (29/47), and secondly introns were absent in the amplified portion of the gene about half of the time (15 /29). When introns were present only one (7 genes), two (1) three (2) or five (1) were found. However, four genes had a more typical eukaryotic intron density with >5 introns, namely, polyketide synthase (18 introns), translation initiation factor 3 subunit 8 (8 introns), small nuclear ribonuclear protein (6 introns), and *psbO* (9 introns). The intron-exon boundaries were, in some cases noncanonical (lacking a GT – AG splicing site). Some of these intron borders revealed an interesting pattern, where the genomic DNA contained 4–6 bases that were identical on both ends of the intron.

### cDNA amplification

Amplifying cDNA templates with a gene specific reverse primer and the general *trans*-spliced leader forward primer should produce products when the *trans*-spliced leader sequence is present at the start of the message and that particular gene is being transcribed. Using this method, cDNAs for 19 genes were successfully amplified. The same primers were also used with genomic DNA in the same PCR master mix to control for misamplification and genomic DNA carryover. In 12 cases inwardly directed gene specific primers produced a PCR product from this batch of cDNA, but when a *trans*-spliced leader primer was used as the forward primer no product was seen. Two genes, *atpH* and *psbO*, were so heterogeneous at the 5′ mRNA ends that direct sequencing of cDNA amplicons was not possible. In these two plastid-targeted genes the different sequences are heterogeneous at the amino acid level in the signal peptide and chloroplast targeting sequence, and the length variation in this region made direct sequencing difficult. These two genes were cloned and 16 of each cDNA were sequenced. Comparisons of *trans*-spliced leader amplified cDNA and genomic amplicons did not show any signs of RNA editing.

### Tandem arrangement in *Karlodinium veneficum*

Two genes, *psbO* and actin were investigated for tandem arrangement in another dinoflagellate, *K. veneficum*. In both cases amplification and sequencing were successful and contiguous assemblies were made between gene copies. The intergenic regions for *psbO* and actin in *K. veneficum* were longer (1490 and 2721 bases, EU742857, EU742856 respectively) and included simple sequence repeat motifs. In the case of actin there was one (7 base), three (10 base) and one (49 base) repeat with from two to 16 repeats in a row; this combined to a total of 407 bases of sequence in a nearly continuous stretch of the intergenic region. The repeats in the *psbO* intergenic spacer were shorter with a three, four and 16 base repeat region covering 152 bases of sequence.

### Actin gene copies in *Amphidinium carterae*

**Figure 3:**
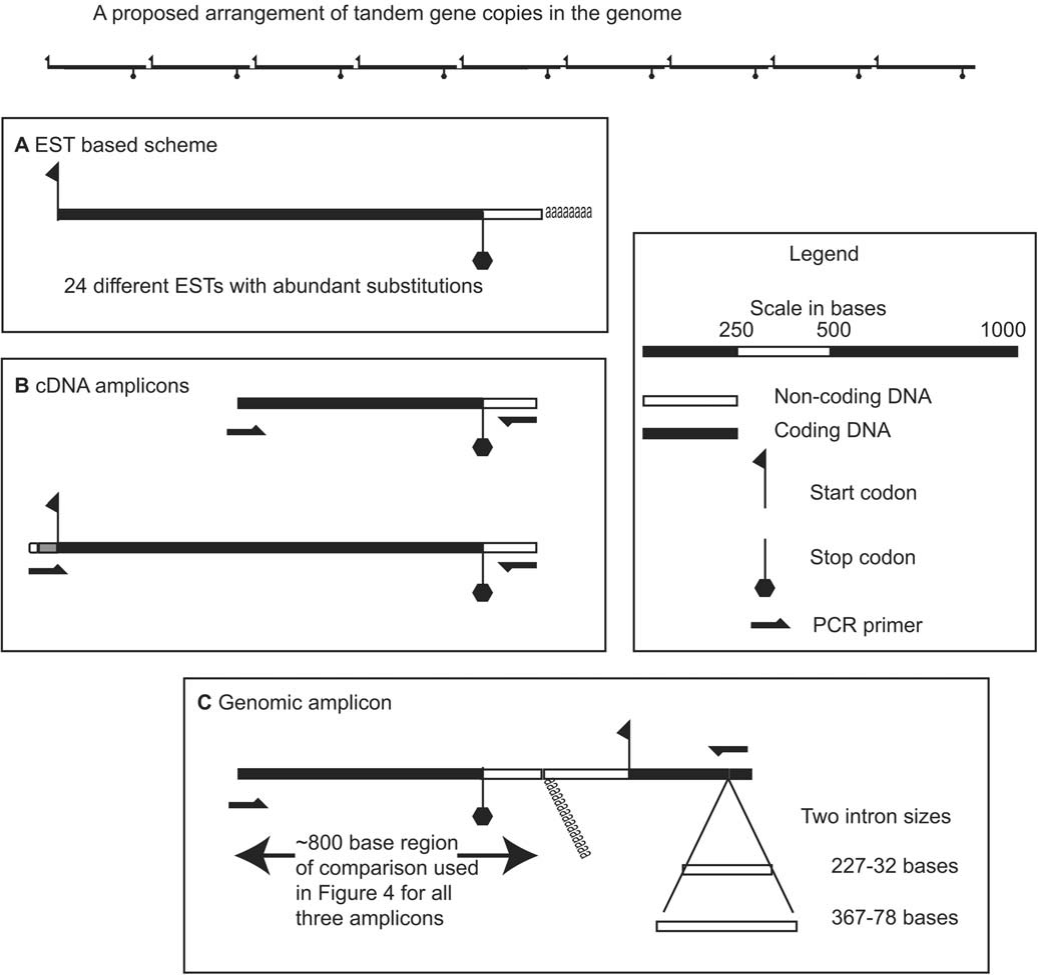
A schematic representation of the different actin amplicons. At the top is a hypothetical arrangement of tandem gene copies in the genome showing a long array of repeated gene copies, with short intergenic regions between them, below are different actin amplicons shown at the same scale. A. An mRNA schematic based on an assembly of 24 different ESTs showing stop and start codons, and the polyA tail. B. Two different cDNA amplicons, the shorter of which was amplified with two gene specific primers (33 were sequenced), and a longer amplicon using the *trans*-spliced leader primer with a gene specific reverse primer (34 were sequenced). C. The genomic amplicon bridging between two adjacent gene copies (46 were sequenced). All schematics are drawn to an equal scale except for the proposed arrangement at the top.

**Figure 4:**
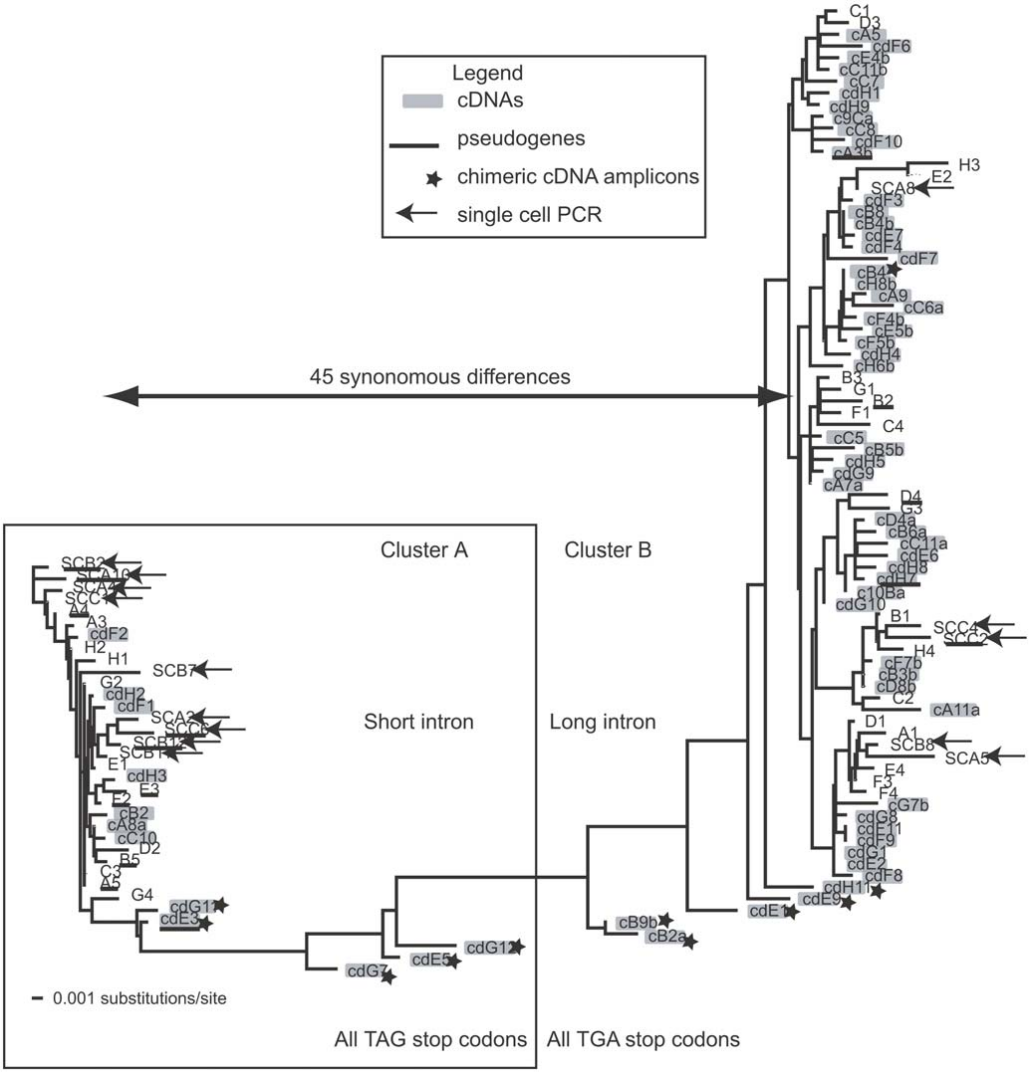
A distance tree using both genomic and expressed versions of the actin gene. The tree was based on an 800 base coding region common to all amplicons (see Figure 3). The two genomic clusters were cleanly divided based on 45 synonymous substitutions over 800 bases, with no intermediate sequences found in the genomic clones (exemplified by the stop codons in this figure). Other features such as intron sequence, and intergenic spacer sequence also sort the two genomic clusters. Multiple pseudogene versions were also found and are underlined. Expressed sequences are marked with colored boxes and were mostly drawn from a single cluster with several chimeric sequences having features of both genomic clusters (marked with a star).

The actin gene was selected for deeper sequencing of genomic tandem, partial cDNA, and full length *trans*-spliced cDNA copies (Figure 3). Working with existing data from ESTs in GenBank as a starting point, BLAST searches and assembly were used to create a cluster of 24 ESTs encoding actin (∼94% nucleotide identity). Other actin genes found in the EST survey that are more distantly related and/or represent different proteins, such as the actin binding protein, were not considered further. While dinoflagellates likely have similar actin binding protein families as other eukaryotes, this study is focused on sequence differences between what appear to be the canonical highly expressed actin genes. Looking at the contiguous assembly of ESTs (Figure 3A), it was quite clear that variation was centered on the third codon position, but there were several small ∼20 base islands of identity between all the sequences. These islands of conservation were used for primer design. PCR products were then generated from cultured genomic DNA, single cell DNA, and two different cDNA amplicons from whole culture RNA (corresponding to B, and C in Figure 3). The resulting sequences share a 800 base region (Figure 3B,C) used to generate the tree (Figure 4).

**Figure 5:**
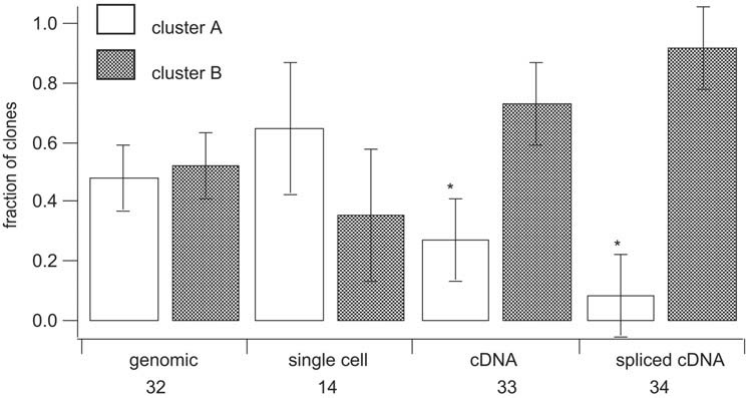
The sorting of different actin clones between the two clusters of copies. The different cluster assignments for genomic culture DNA, single cell genomic, and two different cDNA amplicons (see Figure 3 for arrangement) are shown, asterisks indicate significant differences at p<0.05. Below each category the total number of clones sequenced is given. The cluster A and B assignments refer to the location of the clones on figure 3.

Starting with the genomic amplicons, the sequenced clones were almost equally divided between two clusters (arbitrarily labeled A and B on Figure 4) based on intron length, intergenic spacer size, and sequence differences in both non-coding and coding regions (Figure 3, 4, 5). The term cluster is here used to describe sequences with similar amino acid translations, but dissimilar non-coding regions and is meant to describe variation and similarity between copies of the same gene, rather than differences between members of gene families. Single cell PCR with primers for the genomic actin amplicon followed by cloning and sequencing, produced sequences similar to the amplicons obtained using DNA extracted from cultures (Figure 4). Pseudogenes were common, representing 10 of 46 total sequences with two from cluster A and 8 from B.

Two different actin cDNA amplicons were cloned and sequenced to compare genomic and expressed copies (Figure 3B). A partial cDNA amplicon used the same forward primer as was used for genomic amplification with a reverse primer from a conserved region of the 3′ UTR just before the polyA tail. This reverse primer was identical in all genomic clones. A longer full-length cDNA amplicon used the same 3′ UTR reverse primer combined with a forward primer corresponding to the *trans*-spliced leader sequence to amplify only spliced full-length versions of the actin gene. These different amplicons allow for comparison of the three distinct stages leading to a mature transcript starting from the genome, to a transcript without a *trans*-spliced leader, to a spliced transcript ready for translation.

After sequencing approximately equal numbers of clones from the shorter (33) and longer (34) cDNA amplicons, a more complex pattern of sequence relationships emerged than was seen with the genomic copies. First, most cDNA amplicons clustered within cluster B based both on synonymous substitutions and 3′ UTR sequence, as compared to the equal distribution of genomic amplicons (Figure 4, 5). Ten cDNA sequences, however, appeared to be intermediate between the two genomic clusters (Figure 4). Detailed comparisons along the length of these amplicons revealed a specific substitution pattern. For a large portion of the cDNA sequence the pattern of synonymous substitutions correlated exactly with one corresponding genomic cluster, followed by a switch to the other cluster and maintenance of the character states, or synonymous substitution patterns of that group. Two cDNA sequences appear to switch from one group to another then return to the original sequence group along the length of the sequence.

**Figure 6:**
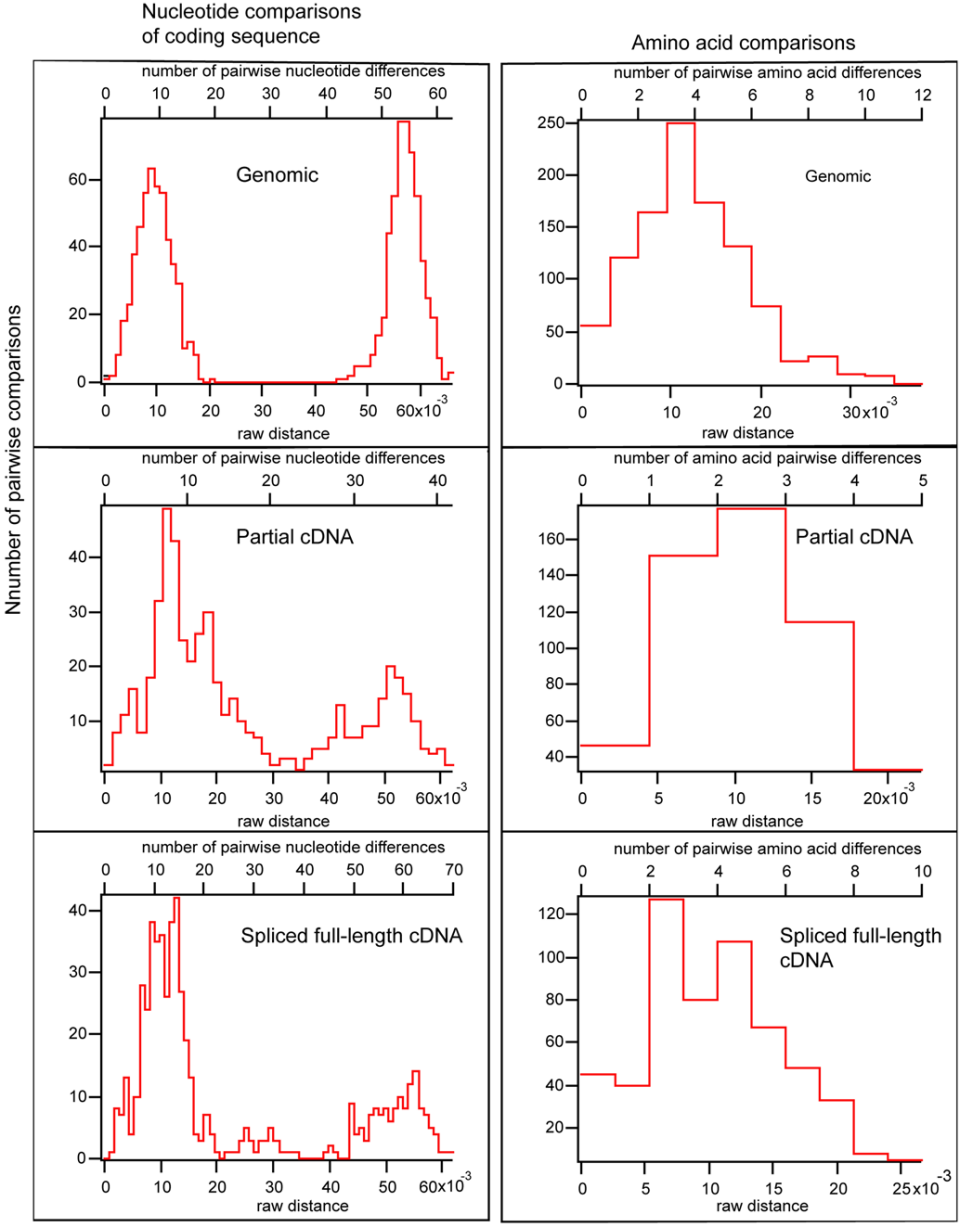
Histograms based on pairwise comparisons of actin gene copies. Based on an alignment of three different actin gene amplicons (diagrammed on figure 3) pairwise distances were calculated comparing the coding regions of three amplicons: genomic, partial cDNA, and full-length *trans*-spliced cDNAs. Each category was treated individually with only the coding regions compared using both nucleotide (left) and amino acid translations (right). The raw distances are the pairwise differences divided by alignment length. In each case, the nucleotide comparisons delimit two different clusters of actin gene copies that are indistinguishable based on amino acid translations.

As a way of summarizing differences between sequence clusters, raw pairwise nucleotide and amino acid differences and distances were calculated based on the coding region for each category of amplicon (Figure 6). Looking first at the genomic copies, the resulting histogram for nucleotide differences shows a bimodal distribution of differences with two distinct peaks at 9 and 54 nucleotides respectively. There were 45 synonymous DNA substitutions in the sequenced portion of the coding region between the two actin genomic clusters (Figure 4), so that comparisons within a single sequence cluster were distributed around a mean of 9 nucleotides and comparisons between groups were distributed around 54 differences. Not a single genomic sequence was found intermediate between these two clusters, although one sequence was found where the first half of the amplicon to the stop codon was from one cluster and the second half from the start codon forward was from the second cluster (EU742751, B2 on Figure 4). Despite the clear distinction between clusters based on nucleotides, no such distinction is found when comparing amino acid differences. With the amino acid differences there is a single maximum of distribution centered around three amino acid differences. When looking at pairwise differences between cDNA amplicons a very similar pattern was seen, with nucleotide differences overwhelmingly found in synonomous (*i.e.* usually third position) sites. In all three cases, from genomic, partial cDNA, and *trans*-spliced cDNA the amino acid differences were distributed around three differences between copies.

**Figure 7:**
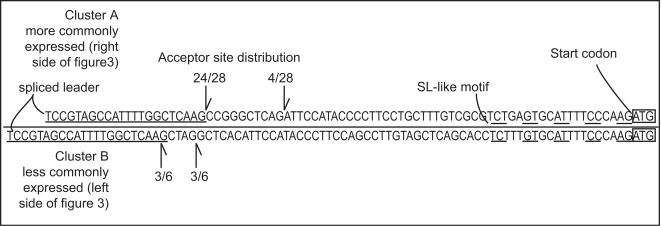
Actin cDNA 5′ UTRs showing *trans*-splice acceptor sites. The 5′ UTRs for the two gene clusters, one above the line and the other below the line, showing the different inferred *trans*-splice acceptor sites. The top sequence represents the more commonly expressed cluster (right side of tree in figure 3) and the lower sequence is rarely expressed (left side of tree in figure 3). Numbers indicate frequency of the splicing pattern in the 34 sequenced cDNAs. The *trans*-spliced leader sequence is underlined, a spliced leader like motif is also underlined (dashed line) just before the start codon (boxed).

Comparing genomic amplicons to *trans*-spliced amplified cDNA sequences revealed the site where the *trans*-spliced leader was added and this was not at a single specific AG acceptor site (Figure 7). The more rarely seen 5′ UTR (6 of 34 clones) had two *trans*-splice sites equally divided between two putative acceptor sites, and the more common 5′ UTR (28 of 34) was predominantly found at one site (24 of 28) with two other rarer locations (4 of 28).

### Polyketide synthase sequencing

**Figure 8:**
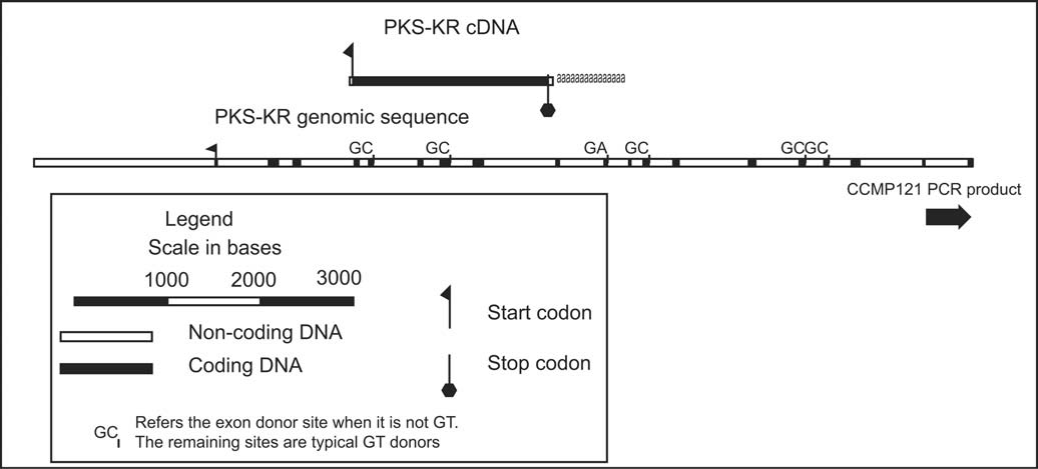
A scaled schematic representation of the polyketide synthase (PKS) genomic and cDNA sequence. Exons, donor sites, and the start codon are shown, the genomic sequence does not extend to the stop codon, but does extend well upstream of the start codon. The location of the amplicon from a second toxin-producing strain of *Amphidinium carterae* CCMP121 is also shown. When atypical intron donors are present the actual dinucleotide donor is shown at the edge of the intron.

The polyketide synthase – ketoyl reductase (PKS) gene was selected for deeper investigation because of the strong contrast in intron density with other genes (Table 1, Figure 8). Initially PCR primers were designed from the fully sequenced cDNA clone, and the genomic complement of the cDNA was sequenced using primer walking. For upstream walking using inverse PCR, the genomic template restricted with both *Kpn*I and *Eco*RI produced amplicons that aligned with the genomic PKS assembly. Primers were then designed for conventional PCR across the restriction site junctions to confirm correct fragment assembly. A total of 1912 bases upstream of the putative 5′ end of the gene were sequenced producing a contiguous assembly of 10,125 bases. A homologous region of the PKS gene was amplified from *Amphidinium carterae* CCMP 121, sequenced and aligned. This region was 100% identical between the two strains based on nucleotide comparisons (500 bases). These two strains produce the same amphidinol toxin (data not shown).

## Discussion

Dinoflagellate nuclei have long been known to be strikingly different from those of other eukaryotes [23], [24]. The loss of nucleosomes [3], [4], high DNA content [1], and modified DNA bases [2] are all features derived within the dinoflagellate lineage. Organellar genome reduction and a history of endosymbiosis create a genome with substantial additions of plastid and mitochondrial genes [10], and potentially genes from other sources as well, such as the form II rubisco [14], [25]. Large gene family size is another feature of dinoflagellates based on a few quantitative studies [15], [16], as well as raw sequence comparison of ESTs [10], [17]. In dinoflagellates, *trans*-splicing has the potential to play a role in gene expression, by creating a pool of fully mature translationally active mRNAs [19], [20]. Transcription factors are also atypical in dinoflagellates [26]. Taken together these features suggest dinoflagellates have a different approach to transcription and gene expression.

By comparing ESTs we detected multiple copies of different genes with substantial variation in synonomous and, in certain cases, non-synonomous sites as well, while confirming that multiple gene copies were expressed [10], [17]. These sequence tags, however, do not shed any light on the genomic context of those genes. The present study provides a preliminary description of the genomic context and variation for a substantial number of genes in *Amphidinium carterae*. However, these results need to be interpreted in the context of the PCR method used, where positive results confirmed by sequencing are significant, but negative results are almost noninformative; further, while the discussion below emphasizes the general nature of these results in dinoflagellates, it is quite possible that specific details may vary between different dinoflagellates, particularly given that *A. carterae*, for a dinoflagellate, has a relatively low DNA content. However, based on targeted gene sequencing in *Lingulodinium polyedrum* [15] and *Symbiodinium* [16], as well as two genes from *Karlodinium veneficum*, the patterns described here probably apply to many dinoflagellates.

### Broad gene survey

The genes found in tandem repeats in this survey were almost all highly expressed (14/16), and almost all highly expressed genes surveyed were found in tandem repeats (14/15; Table 1; Figure 1). Expression level was based on abundance in an EST survey of ∼3400 sequences [10]. The majority of genes surveyed were not demonstrated to be in tandem arrays. In general, negative PCR results are not informative, but in the context of this project there is a pattern of success with highly expressed genes and failure with less highly expressed genes. These results contrast with internal amplification using inwardly directed primers, and with cDNA amplification. Internal primers worked in the majority of cases and success or failure was not correlated with expression level. While we show highly expressed genes are in tandem arrays, the methods used here cannot prove that less highly expressed genes are not. The size range of amplicons was up to ∼4.5 kilobases for elongation factor 2 amplicon, suggesting the limits on the range of PCR under these conditions. Less highly expressed genes may have a different genomic context such as larger intergenic regions, or other genes may be interspersed between copies. For the basic nuclear protein there was one clone with a different pattern than a simple tandem repeat. In this clone another basic nuclear protein variant was interspersed between copies of the first protein. The small size of this protein (<100 amino acids) made amplification across two copies relatively easy. Clearly then the PCR test used here would be biased for tandem repeat genes with short intergenic regions (Figure 1) and would not detect other gene arrangements.

For highly expressed genes intergenic regions were compact with only a small region completely excised from the mRNA (Figure 1). The short 5′ and 3′ UTRs were relatively uniform in size. As a further test two genes, actin and *psbO*, were amplified from tandem arrays in another dinoflagellate *Karlodinium veneficum*. While the overall pattern of tandem repeats held, the intergenic spacers were larger (1.5 and 2.7 kb) and contained simple sequence repeats. The intergenic spacers of luciferase have also been shown to contain repeats [11], [27], so it seems as if the absence of simple sequence repeats in the intergenic spacers of *A. carterae* is the exception in this small sample size.

Intergenic regions allowed for comparison of the cDNA ends with their genomic complement at both the 5′ and 3′ ends. The polyadenylation site in the cDNA is a ‘miniature’ polyA region in the genomic version with 3–5 adenine residues often followed by a G or C (Figure 2). Dinoflagellates lack the canonical AAUA polyadenylation signal [28], making the polyadenylation signal unclear. The pattern of short polyA stretches in the genomic DNA was also found in the two *K. veneficum* genes examined, and at the end of the 3′ UTRs for *L. polyedrum* peridinin-chlorophyll a-protein gene and luciferin binding protein, suggesting a general pattern in dinoflagellates. The polyA genomic site may be both a signal for polyadenylation and/or a byproduct of duplication. On the 5′ end, while the *trans*-splice acceptor site is clearly an AG, there are no simple primary sequence consensus features that can be easily described, and there is some degree of flexibility in the exact AG acceptor (see below; Figure 7).

Another feature suggestive of an RNA intermediate in a few genes in *A. carterae* is a *trans*-spliced leader like sequence preceding the start codon in the genomic sequence. In the case of the peridinin-chlorophyll a-protein gene the genomic sequence is at the exact *trans*-splicing site, making it possible for a message to be made that contains the spliced leader without *trans*-splicing. In other cases, although the *trans*-spliced leader-like sequence precedes the start codon, it is not an exact match (Figure 7). These two features, a mini polyA region and an spliced leader motif, seem to be a common feature of highly expressed genes, almost as if they are amplified *via* reverse transcription. The low intron density of highly expressed genes also fits well with this possibility.

### Splicing survey

Testing all cDNAs for the presence of a *trans*-spliced leader revealed that it was present in approximately two thirds of genes for which cDNA could be detected (Table 1). The distribution of *trans*-splicing is also strongly correlated with expression level, with almost all (12/15) highly expressed genes *trans*-spliced. The only two tandem repeat genes not shown to be *trans*-spliced may not have been present in the cDNA used, based on negative results with gene specific primers. However, six genes that were not shown to be in tandem arrays and were not highly expressed, were shown to be *trans*-spliced. Based on these results it is tempting to speculate that some messages are not *trans*-spliced, but it is important to note that the PCR test is not significant when negative. Ultimately comparisons of many ESTs derived from a *trans*-spliced leader amplified library and a non-amplified library and RACE on selected mRNAs should help shed light on these questions.

### Categories of genes in the dinoflagellate genome

Therefore, there are potentially at least two different types of gene arrangement (tandem repeat and not) and potentially at least two different types of expression (*trans*-spliced and some alternate form) and these two categories may not be completely correlated, although tandem repeat genes are almost always *trans*-spliced. We propose two general categories of genes in the dinoflagellate genome: highly, and moderately expressed. The highly expressed genes are found in tandem repeats with low intron density, and the messages from these genes are *trans*-spliced. High copy genes are present as many slightly different gene copies, as evidenced by sequence differences in intergenic regions, introns, and both coding and non coding regions of cDNAs and ESTs. The moderately expressed genes are by definition less well represented in EST surveys, and so appear to be single copy, and also have significantly higher intron density. Only a few genes were found with more than three introns; four genes including the PKS, *psbO*, a translation initiation factor, and a small nuclear ribonuclear protein were more intron dense (>5 introns; Table 1). These features could be used to create a category of genes with high intron density, lower expression level and putatively low copy number in the genome. However, *psbO* is a tandem repeat gene, and is present at a relatively high copy number based on polymorphisms between different EST and cDNA copies, but is intron rich. We selected two genes, actin and a PKS gene, as examples of the two categories, and did deeper sequencing on them.

### Actin gene copies

Actin is used here as an example of a highly expressed tandem copy gene largely because it is a familiar and easily defined gene. The differences between copies described here are relatively small at the amino acid level (∼1% difference, Figure 6), and do not rise to the level of the actin related proteins found in other eukaryotes [29]. Two actin genomic clusters were described here, but EST data suggest more diversity in this gene family and sequencing of cloned products was clearly not exhaustive within the two clusters sampled, even across the full alignment of 113 sequences. One explanation for actin copy diversity could be that the culture was not clonal, or that individual cells were rapidly diverging within the culture, but single cell PCR results corresponded well with that seen in culture genomic DNA (Figure 4,5).

The differences between the two gene clusters are present at two different types of sites. First, the non-coding introns and intergenic spacers were well conserved within clusters, with little length variation or primary sequence divergence. Second, synonomous sites in the coding region were also strongly conserved within clusters making it possible to define the clusters solely on the basis of which stop codon was found, for example (Figure 4). The correlation between non-coding sequence and cluster allegiance was absolute, suggesting a concerted evolutionary process within but not between, clusters of actin copies. These results fit well with the results of previous studies. In one case, the *L. polyedrum* peridinin-chlorophyll a-protein gene, only one absolutely identical tandemly repeated sequence is present in the genome with thousands of copies, based both on genomic and expressed versions of this gene [15]. The many thousands of identical copies of the peridinin-chlorophyll a-protein gene may undergo concerted evolution like an rRNA repeat while other highly expressed genes in *L. polyedrum* were found to have multiple different sequences [10]. In contrast, in *Symbiodinium* sp. the peridinin-chlorphyll a-protein gene had many different amino acid variants and a lower copy number [16]. As speculation, perhaps at a certain copy number a gene conversion threshold is crossed so that the copies are more efficiently constrained to a single sequence.

The pattern of concerted evolution in the neutral sites of the different actin gene clusters contrasts with the variation in amino acid sequence between copies. The amino acid variation does not correlate with cluster allegiance, so that amino acid based phylogenies of the different actin gene copies do not resolve the two clusters (data not shown). The pairwise distance (or difference) histograms using coding nucleotides and amino acids demonstrate this paradox between amino acid and nucleotide differences (Figure 6). There are clear constraints on both nucleotide and amino acid sequence, but the proscribed degree of variation (in nucleotides within clusters, and in amino acids between all copies) is neatly distributed around an average of three amino acid differences between gene copies. We then thought to look at differences in expression of the different actin copies to see if any patterns would emerge.

### Expression of different actin copies

Previous results indicate transcript levels for circadian controlled proteins do not change with protein levels in dinoflagellates e.g. [18] and the recent discovery of *trans*-splicing seems to provide an explanation [19], [20]. One possibility is that only successfully *trans*-spliced messages can be translated, so that expression may be at least partially controlled by *trans*-splicing rather than transcription. Sequencing multiple versions of actin from the genome, the transcriptome, and the *trans*-spliced transcriptome allowed assessment of bias at these different levels between the two clusters of genomic copies (Figure 5). Two classes of cDNAs were compared, full length *trans*-spliced versions of actin amplified exclusively from the *trans*-spliced transcriptome, and a shorter amplicon, representing the total pool of actin message amplified with only gene specific primers. It is completely possible that the total pool of actin message is *trans*-spliced, in which case there would not be a detectable difference between the full length *trans*-spliced copies, and the partial copies. Based on the data presented here there is significant bias in both the transcriptome and the *trans*-spliced transcriptome when compared to the genome, but this bias is not significant when comparing the spliced and total transcriptome (Figure 5). These results are quite preliminary, since the assessment is based exclusively on PCR results, although the primer sets were carefully chosen to be invariant between clusters to minimize artifacts. Using quantitative PCR seems like a logical next step but, given the sequence variation between gene copies and the constraints on primer design, it is not clear if the results would be valid. These results suggest selection of a specific cluster of gene copies for expression, either at the level of transcription or *trans*-splicing or both.

The site of *trans*-spliced leader addition in actin cDNAs indicates another pattern of diversity. While most cDNAs were spliced at one site, it is clear that the *trans*-splicing site is not an exact AG acceptor, but is rather possible at two or more sites per cluster (Figure 7). This same pattern of diversity was seen in other cloned *trans*-spliced leader cDNAs, such as *psbO* and *atpH*. These results suggest some flexibility in *trans*-splicing, and thus a potential way of modulating spliced leader addition based on specific features of the intergenic region. In other words, the bias favoring one cluster in the transcriptome may be the result of a more easily *trans*-spliced intergenic region in one cluster of copies.

Another intriguing result is the presence of actin cDNA sequences intermediate between the two families, not seen in the genomic clones (Figure 4). If genomic sequencing is representative, then in the genome there are no actin copies intermediate between the two families, or in other words, all the synonomous substitutions are conserved within clusters in the genome. An artifact such as PCR recombination in the cDNA amplification may be at work, because it seems there is higher diversity in expressed sequences than is found in the genome. The cluster allegiance of the cDNAs appear to shift at discrete points along the sequence, suggesting a splicing or PCR recombination event could be occurring between copies of the same gene at favored sites. It almost seems as if an alternate splicing event is occurring between the heterologous sequences, resulting in an alternatively spliced chimeric cDNA. Certainly proof of this would require both exhaustive sequencing of genomic copies and some clear way of avoiding PCR recombination.

### Moderately expressed gene category

The results of actin sequencing showed many tandemly arranged gene copies with low intron density; EST data and single gene studies indicated this gene category may include many highly expressed genes. The PKS gene most strongly contrasts with this pattern due to high intron density and low expression (Figure 8). As a test for a possible culture contamination, a homologous region was sequenced from another strain of *A. carterae* CCMP121 (other *Amphidinium* species did not amplify). Either there is a similar contaminant, or the gene is genuinely present in the dinoflagellate genome. Amplification of the PKS cDNA was possible using a pair of gene specific primers, so the transcript was present in the cDNA pool, but combining a *trans*-spliced leader primer with a gene specific primer did not successfully amplify this gene, a result that suggests, but does not prove, that the PKS gene may not be *trans*-spliced. Direct sequencing of PCR products for PKS was simple, whereas with the actin gene direct sequencing revealed DNA polymorphisms as ambiguous base calls (double peaks) and was impossible in introns or intergenic regions where insertions and deletions made sequencing impossible. Upstream sequencing from the PKS gene using inverse PCR did not indicate any other recognizable copy of this gene was nearby. Thus, while there is still the possibility that the PKS genes could be found in more broadly spaced tandem repeats, this gene does not appear to follow the pattern of the more highly expressed genes with compact intergenic regions. The arrangement of this genomic sequence is reminiscent of the descriptions in [30], but these sequences have not been deposited in GenBank. Taken together results suggest, but cannot prove, that the PKS gene is not present as a high copy number tandem array gene with low intron density, and thus help to define a potentially single copy, moderately expressed, intron dense class of genes.

### Comparison to other systems

There are evident analogies between dinoflagellates and the trypanosomes, despite their allegiance to distinct distantly related lineages. Trypanosomes are parasitic protists where mRNA maturation, and ultimately translation requires *trans*-splicing, rather than controlling expression by transcription. However, in trypanosomes most genes are present, not in replicated arrays, but as a series of different genes tandemly arranged on a single chromosome. Transcription initiation occurs from a specific site on each chromosome with ‘polycistronic’ RNA intermediates that are in turn *trans*-spliced and polyadenylated based on splice site recognition [31]–[33]. Applying these concepts to dinoflagellates would imply that transcription initiation from hypothetical long tandem gene arrays may occur at a specific flanking site. This idea could help explain the lack of canonical promoters in dinoflagellates, and to some extent the unlinking of expression, as measured by northern blots or microarrays, and protein level [18]. The amount of transcript could be proportional to the number of gene copies, with more highly expressed genes having a higher copy number. Control of translation could occur during processing of the message including: *cis* splicing of individual gene copies and introns, *trans*-splicing of the spliced leader, and polyadenylation. Clearly the analogy between trypanosomes and dinoflagellates is intriguing, but the development of these two unusual genomic systems appears to be yet another example of convergent evolution.

In many eukaryotes the genome is amplified by DNA divisions without cytokinesis during specific developmental stages [34], [35]. In dinoflagellates it remains unclear if the high DNA content is related to polyploidy or a process of endoduplication, but if so, the unduplicated intermediate has not been observed. The unusual process of ‘cyclosis’ seen after meiosis in dinoflagellates does not seem to be accompanied by genome duplication, in so far as this process has been observed at the level of genome size [36], but it is possible to imagine interlaced networks of tandem gene copies somehow contributing to chromosomal condensation. This is akin to the way that long repeats of genes inserted into animal genomes are more likely to be condensed into heterochromatin and thus suppressed [37]. This comparison is a bit of a stretch since dinoflagellates really do not have the basic nucleosome unit found in other eukaryotes, but perhaps at some level the chromosomes of dinoflagellates are structured based on stretches of tandem repeat DNA. Comparisons can also be made with the arrays of histone genes in animals where many tandem arrays are found in the genome and the individual genes are not interrupted by introns. However, histone arrays are usually arranged as a quintet of the different histone genes, often with some genes encoded on the opposite strand, so this comparison is not precisely the same [38].

### Conclusions

The ‘black box’ of the dinoflagellate genome can now be divided on several levels. Most striking is that highly expressed genes are present at a high copy number in repeated tandem arrays, and *trans*-splicing of these genes was readily detected. Many of these high copy genes probably have gene clusters containing many subtly different sequences for what is ultimately functionally the same protein, as was demonstrated for the actin gene in *A. carterae*, and has been seen in EST surveys as well. There is a second class of genes with high intron density and apparently lower copy number that may be not be *trans*-spliced. These categories of genes would be interesting to approach by screening a large-insert library, but this library would likely prove to be frustrating when tandem repeat genes were cloned. Even if such clones were stable, sequencing of multiple tandem repeat gene copies would require precise base calling, and primer walking strategies would likely not work. So, while a large insert library is the only practical way to represent the entire genome, such a library is likely to be subject to bias that will make it not fully representational. Other challenges with genome libraries from dinoflagellates include modified DNA bases and bacterial contamination. The genomic characteristics shown here for *A. carterae* have to be demonstrated for a larger sample size of dinoflagellates, including representatives from many different lineages to determine if tandem gene arrangement and high copy number are present across the entire lineage. Preliminary results from genome sequence of the outgroup ‘proto-alveolate’ *Perkinsus marinus*, suggest tandem arrays of the sort described here are not present (www.tigr.org/tdb/e2k1/pmg/). The results of broader sequencing efforts would also help to understand how so many different variable copies of these genes have evolved and are maintained in the genome.

### Relevance to hosting endosymbionts

Because dinoflagellates have adopted so many different plastid types [39], they are assumed to have an effective gene transfer mechanism. Thousands of nuclear encoded genes are required to maintain a plastid, since most cyanobacterial genes have migrated from the plastid genome [10], [40]. In dinoflagellates, this process has proceeded to an extent not seen in other eukaryotes, with tens of genes retained on DNA minicircles thought to be relics of the plastid genome rather than the hundreds of genes found in other plastid genomes [9], [10], [21]. One hypothesis would be that dinoflagellates are able to incorporate foreign genes more easily because they have an expression system that is regulated differently from other eukaryotes. The logic behind this argument is that for gene transfer to be successful the gene has to be not only incorporated into the genome, but it also appropriately regulated and transcribed. If dinoflagellate gene regulation avoids promoters, and instead relies on *trans*-splicing and gene dosing, a potentially significant barrier to gene transfer is removed. The apparent amplification of gene copies shown here suggests an inherent flexibility in the genome. *Trans*-splicing could provide a unified mechanism for incorporating mRNAs into the pool of translatable messages. In the genomic survey presented here, representatives of all classes of dinoflagellate genes (as defined by [41]) were sampled (Table 1), including putative host genes (actin), those more recently transferred from the plastid genome (*atpH*), and genes that are directed to the plastid but nuclear encoded in eukaryotes such as plastid GAPDH and *psbO*. However, gene origin did not display a clear relationship to genomic category, with expression level explaining the data most clearly. Thus, highly expressed plastid associated genes were found in tandem arrays and were spliced, while less highly expressed plastid genes such as glutamate semialdehyde synthase and fructose 1,6 biphosphate aldolase did not seem to be in tandem repeat arrays (Table 1). On the whole, dinoflagellates likely have lowered barriers to gene transfer, but much work remains to explain exactly how dinoflagellates incorporate new genes.

## Methods

*Amphidinium carterae* strain CCMP1314 was grown in natural seawater with a salinity of 32 supplemented with f/2 nutrients at 20°C with 100 mE/m^2^ s^−^ light [42]. Cells were harvested by centrifugation, the resulting pellet frozen in liquid nitrogen, and extracted using a CTAB detergent method [43]. DNA quantity and quality were determined by spectrophotometry and agarose gel electrophoresis. For the cultures of *A. carterae* CCMP 121 and *Karlodinium veneficum* CCMP 2282, culturing and DNA isolation was performed in the same manner. For single cell PCR individual cells of *A. carterae* were selected from cultures using a drawn glass pipette on an inverted microscope magnified 100 times. Individual cells were washed three times in minimal volumes of sterile media, and placed in 9 µL of water. The cells in water were kept on ice until 21 cells were collected into individual tubes and subsequently frozen at −20°C. 10 µL of PCR master mix (described below) was added and PCR cycling performed.

Sequences were selected from the *A. carterae* EST project, largely from readily identified sequences with significant blast hits, but also including a mitochondrial gene (*coxIII*), and several highly expressed but difficult to identify genes such as an ‘Ectocarpus silicosus virus’ (ESV) like gene with a polysaccharide binding motif (Table 1). Forty seven unique sequence entities, either contiguous assemblies of ESTs or single sequences, were selected. Primers were designed to amplify both in a typical inward direction, and in a reverse orientation between gene copies using the Primer3 software in batch mode (table of primers available on request). For between copy primers the sequences were cut in half and pasted in a reverse orientation to generate properly directed primer sets. PCR was performed using a MJ gradient thermocycler with a range of three or more temperatures from 55–65°C annealing with a 15 s dissociation stage at 94°C, 15 s annealing, and 60 s extension at 72°C for 40 cycles in a PCR containing 500 mg/mL BSA (Sigma A2053), 50 mM Tris HCl pH 8.3 and 3 mM Mg with 0.12 units of Promega Go-Taq in a total volume of 20 µL. Products were detected on a 1% agarose gel using ethidium bromide staining and were precipitated with a 20% w/v polyethylene glycol (mw 8000), 2.5 M NaCl solution, resuspended in water, and sequenced using ABI BigDyev3.0 terminator chemistry. Direct sequencing was used for inwardly directed products and most cDNA amplicons, but was not useful for most intergenic regions, likely due to size differences in the products. In those cases, cloning using the pGEM-T vector, ligase, and competent cells from Promega was used. The resulting colonies were screened by PCR with M13 primers using template prepared by heating a single colony in 50 µL of TE to 94°C for 5 min., followed by centrifugation at 1000 rpm for 5 min in a clinical centrifuge. The PCR products corresponding to cloned inserts were examined on a gel, purified by PEG precipitation, and sequenced as described above.

For inverse PCR 1 µg of total DNA was restricted with *Eco*RI, *Apa*I, or *Kpn*II overnight. The DNA was then diluted to a concentration of 10 ng/mL and ligated overnight at 15°C using 3 units of T4 ligase (Promega) in the supplied buffer. Primers were designed to allow for nested PCR, and the Expand Long Template PCR system from Roche was used for amplification.

For tandem array amplification from *Karlodinium veneficum* CCMP 2282 DNA was isolated using the CTAB method, and primers were designed to amplify tandem repeats for the genes actin and *psbO*.

For cDNA PCR amplification total RNA was prepared from 50 mL of pelleted *A. carterae* culture using Tri-Reagent (50 mL×80,000 cells/mL; Sigma T9424). The total RNA was reverse transcribed using a matrix of three different RNA input amounts (1, 3 and 9 µg per reaction) and three different reverse transcription temperatures (37, 42 and 50°C) with Invitrogen superscript III and the buffer supplied with the enzyme. The quality of the cDNA was then tested using PCR with GAPDH primers and examining the products on a gel. The reactions with 1 and 3 µg of input RNA worked well at all temperatures and were diluted 10 fold with water for use in PCR using both a spliced leader primer with gene specific reverse primers, and using standard forward and reverse gene specific primers. The products from these reactions were compared to those using genomic DNA to confirm amplification from cDNA and sequencing was used to confirm the authenticity of the products and exact splicing location.

Sequences were trimmed with Sequencher 4.7 (GeneCodes) to remove vector and ambiguous sequence and compared to cDNAs using contig assembly, blast, and manual alignments as necessary to define intron/exon boundaries, start and stop codons, splice sites and polyadenylation sites. Sequence editing was performed with Sequencher 4.7, and alignments were manipulated in MacClade 4.08 [44]. PAUP* version 4b10 [45] was used to calculate pairwise distances and create phylogenetic trees. Histograms were constructed using Igor Pro graphing software (Wavemetrics).
